# Dopaminergic Inhibition of Na^+^ Currents in Vestibular Inner Ear Afferents

**DOI:** 10.3389/fnins.2021.710321

**Published:** 2021-09-09

**Authors:** Frances L. Meredith, Katherine J. Rennie

**Affiliations:** ^1^Department of Otolaryngology – Head & Neck Surgery, School of Medicine, University of Colorado, Aurora, CO, United States; ^2^Department of Physiology & Biophysics, School of Medicine, University of Colorado, Aurora, CO, United States

**Keywords:** calyx, semicircular canal, crista, hair cell, sodium channel

## Abstract

Inner ear hair cells form synapses with afferent terminals and afferent neurons carry signals as action potentials to the central nervous system. Efferent neurons have their origins in the brainstem and some make synaptic contact with afferent dendrites beneath hair cells. Several neurotransmitters have been identified that may be released from efferent terminals to modulate afferent activity. Dopamine is a candidate efferent neurotransmitter in both the vestibular and auditory systems. Within the cochlea, activation of dopamine receptors may reduce excitotoxicity at the inner hair cell synapse via a direct effect of dopamine on afferent terminals. Here we investigated the effect of dopamine on sodium currents in acutely dissociated vestibular afferent calyces to determine if dopaminergic signaling could also modulate vestibular responses. Calyx terminals were isolated along with their accompanying type I hair cells from the cristae of gerbils (P15-33) and whole cell patch clamp recordings performed. Large transient sodium currents were present in all isolated calyces; compared to data from crista slices, resurgent Na^+^ currents were rare. Perfusion of dopamine (100 μM) in the extracellular solution significantly reduced peak transient Na^+^ currents by approximately 20% of control. A decrease in Na^+^ current amplitude was also seen with extracellular application of the D2 dopamine receptor agonist quinpirole, whereas the D2 receptor antagonist eticlopride largely abolished the response to dopamine. Inclusion of the phosphatase inhibitor okadaic acid in the patch electrode solution occluded the response to dopamine. The reduction in calyx sodium current in response to dopamine suggests efferent signaling through D2 dopaminergic receptors may occur via common mechanisms to decrease excitability in inner ear afferents.

## Introduction

The inner ear houses the cochlea and vestibular system where signals relevant to auditory and vestibular perception are processed by hair cells and carried to the brain by their companion afferent nerve fibers. In mammals outer and inner hair cells are found in the cochlea, whereas type I and type II hair cells populate the sensory epithelia of the vestibular organs. Although each hair cell type is tailored toward its specific sensory purpose, all vertebrate hair cells transduce displacement of their mechanosensitive apical hair bundles into a receptor potential which regulates release of the neurotransmitter glutamate onto unmyelinated afferent terminals. Auditory and vestibular signals are conveyed in the VIII^*th*^ nerve to the brain as action potentials in afferent neurons which have diverse properties in terms of their spontaneous action potential firing rates, thresholds and frequency responses (Goldberg, 2000; Eatock and Songer, 2011). The cochlea and vestibular organs also receive substantial efferent innervation from the brain. In the mature cochlea, medial olivocochlear efferents terminate on outer hair cells and lateral olivocochlear neurons make synapses with afferent nerve fibers beneath inner hair cells (Warr and GuinanJr., 1979). In mature vestibular epithelia, efferent fibers emanate from the brainstem to make synapses with type II hair cells and with afferent dendrites including the outer aspects of large calyx terminals surrounding type I hair cells (Wersall, 1956). The location of efferent synapses on afferent dendrites postsynaptic to hair cells supports a role for efferent modulation of glutamatergic transmission. For efferent terminals that synapse directly with type II hair cells and outer hair cells, acetylcholine acts via α9-containing nicotinic receptors to activate small conductance calcium-activated K^+^ (SK) channels and hyperpolarize the membrane thus serving an inhibitory role (Fuchs and Lauer, 2019; Poppi et al., 2020). In turtle crista, efferent release of acetylcholine can also produce excitation through the muscarinic inhibition of K^+^ currents mediated by KCNQ channels in calyx-bearing afferents (Holt et al., 2017; Lee et al., 2017).

Although acetylcholine is considered to be a major efferent transmitter in both the cochlea and vestibular periphery (Fuchs and Lauer, 2019; Poppi et al., 2020), other transmitters, including dopamine, may also function as neuromodulators in hair cell systems. Previous work has provided evidence for dopamine receptors in hair cell organs including frog crista, fish lateral line and saccule and rodent cochlea and vestibular otolith organs (Drescher et al., 2010; Maison et al., 2012; Toro et al., 2015; Perelmuter et al., 2019). In frog semicircular canal afferents, application of dopamine and dopaminergic agonists decreased the firing rate of action potentials (Andrianov et al., 2009). Similarly, dopamine reduced action potential firing rate in auditory afferents of guinea pig and rat cochlea (Oestreicher et al., 1997; Ruel et al., 2006; Wu et al., 2020). Sound exposure increased dopamine production in efferent neurons of mice suggesting a neuroprotective role (Maison et al., 2012; Wu et al., 2020). Together these studies support a role for dopaminergic signaling in diverse hair cell systems either through direct synaptic action or via paracrine signals.

The role of efferents in the peripheral vestibular system is poorly understood. To date, direct effects of dopamine on afferent neurons in mammalian vestibular epithelia have not been ascertained. To address this we isolated calyx terminals from semicircular canal organs of gerbils and tested the effects of dopamine on their Na^+^ and K^+^ currents. After finding a reduction in transient Na^+^ currents in response to dopamine we further investigated underlying receptors and possible signaling pathways. We demonstrate a reduction in transient Na^+^ currents in response to dopamine that appears to be mediated by D2 receptors, suggesting that efferent-mediated dopamine release may lead to decreased firing in vestibular afferents.

## Materials and Methods

### Tissue Preparation

Mongolian gerbils (*Meriones unguiculatus*) of both sexes were used at postnatal days (P)13 to P33. Intraperitoneal injections of ketamine (70 mg/kg) and xylazine (3 mg/kg) in sterile saline were used to induce anesthesia with supplemental doses given when necessary. Following decapitation the cristae of the semicircular canals were dissected out and immersed in Leibovitz’s L-15 medium (pH 7.4–7.45, osmolality 300–305 mosmol/kg H_2_O solution). Procedures were in accordance with NIH guidelines and protocols approved by the University of Colorado’s Institutional Animal Care and Use Committee.

Hair cells and calyces were mechanically separated from cristae with procedures similar to those described previously (Rennie and Streeter, 2006; Dhawan et al., 2010). Briefly the mechanical dissociation involved drawing a fine probe across the crista which was immersed in extracellular solution in the recording dish. For slice recordings (Figure 4B) ampullae were embedded in a low gelling temperature agarose and transverse slices (100 μm) cut through the end organ on a vibrating blade microtome (Leica VT1200 S, Leica Biosystems) (Meredith and Rennie, 2018, 2020). The cell recordings obtained for slice data in Figure 4B were reported previously (Meredith and Rennie, 2020).

### Electrophysiological Recordings

Cells were visualized in the recording dish with a water immersion objective (X40) and differential interference contrast (DIC) optics on an Olympus microscope (BX51). Isolated cell pairs of single type I hair cells enveloped by calyx terminals or calyces within slices were selected for recordings. Electrodes were pulled from electrode glass (PG165T, Warner Instruments, Hamden, CT, United States) on a multistep horizontal puller (P-97 Sutter Instruments, San Rafael, CA, United States) and electrode tips were polished by heat using a Narishige MF 830 microforge (Narishige International United States, East Meadow, NY, United States). SYLGARD 18 (Dow Corning, Midland, MI, United States) was applied close to the tip of each electrode to reduce stray capacitance.

Calyx terminals express several different types of voltage-gated conductances including Na^+^ currents (Meredith and Rennie, 2016). For experiments investigating the effect of dopamine on K^+^ currents the electrode solution contained (in mM): KF (115), KCl (10), NaCl (2), HEPES (10), D-glucose (3), MgCl_2_ (2), EGTA (10), pH 7.4 adjusted with KOH (27 mM). For all other experiments the electrode solution contained (in mM): CsF (120), CsCl (10), NaCl (2), HEPES (10), D-glucose (3), MgCl_2_ (2), EGTA (10), pH 7.4 adjusted with CsOH (24 mM). To further isolate Na^+^ currents from other conductances, recordings for investigating resurgent currents (I_*NaR*_) were obtained in an extracellular solution containing in: (mM): NaCl (120), CsCl (5.4), MgCl_2_ (2.5), tetraethylammonium Cl (30), CaCl_2_ (1.3), glucose (10), HEPES (5). For all other experiments the external solution was Leibovitz’s L-15 medium.

Electrode resistance was 2.3–5 MΩ prior to seal formation on calyces. Recordings were made at room temperature (21 – 24°C) with voltage protocols designed to probe for transient (I_*NaT*_) and resurgent (I_*NaR*_) Na^+^ currents. Data were acquired using pCLAMP 10 software with a patch amplifier (Axopatch 200B, Molecular Devices, Sunnyvale, CA, United States) connected to a PC via an AD converter (Digidata 1440A, Molecular Devices, Sunnyvale, CA, United States). Signals were low-pass filtered online at 5 or 10 kHz and sampled at 20–50 kHz. Correction for liquid junction potentials was made in the analysis.

Dopamine was dissolved in deionized water or L-15 on each experimental day to make a 10 mM stock solution which was then added to L-15 for a final concentration of 100 μM. In experiments with Cs^+^-based electrode solutions dopamine solutions also contained 100 μM ascorbate. Most chemicals including dopamine hydrochloride were obtained from Sigma Aldrich. Okadaic acid, ascorbic acid, quinpirole hydrochloride and eticlopride were from Tocris Bioscience. The external solution was perfused using a peristaltic pump (Gilson) at a flow rate of 0.5-1.0 ml/min. Drugs were typically perfused for a minimum of 4-9 min before measurements of drug effects on Na^+^ currents were taken. The length of the wash (return to control solution) was between 6 and 10 min.

### Data Analysis

Clampfit 10 (Molecular Devices) and Sigmaplot 11 (Systat Software, San Jose, CA, United States) were used to analyze voltage clamp data. Mean values ± SD are reported and values were compared with paired *t*-tests.

## Results

### Dopamine Reduces Na^+^ Currents but Not K^+^ Currents in Isolated Calyx Terminals

**FIGURE 1:**
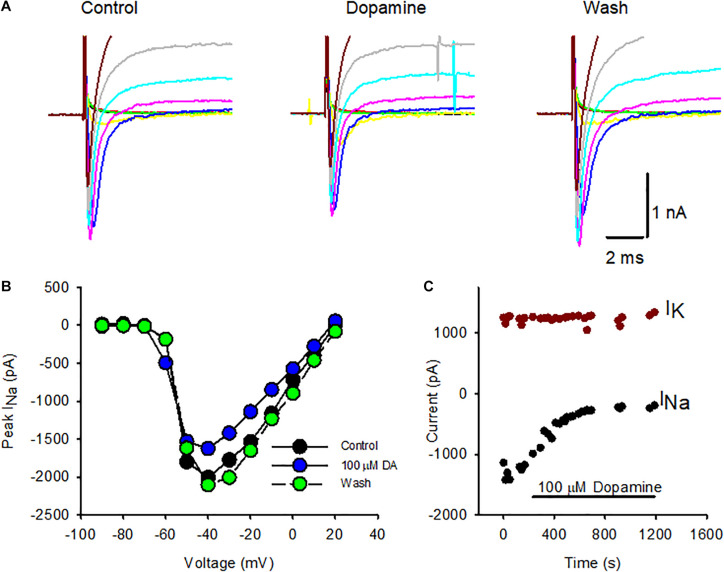
Effect of dopamine on Na^+^ and K^+^ currents in isolated calyx terminals. **(A)** Whole cell currents in an isolated calyx in response to a series of voltage steps from –90 to –10 mV following a prepulse to –130 mV. Transient Na^+^ currents were reduced in response to application of 100 μM dopamine and recovered following washout. **(B)** Current-voltage plot comparing peak I_*NaT*_ at each voltage step in control conditions (black circles), during dopamine (DA) perfusion (blue circles, 6 min of perfusion) and following washout (green circles, 10 min of perfusion). P24 calyx, female. **(C)** I_*NaT*_ and steady state potassium current (I_*K*_) were measured during perfusion of 100 μM dopamine to an isolated calyx. Peak I_*NaT*_ was measured at successive voltage steps to –40 mV following a prepulse to –130 mV to remove Na^+^ channel inactivation (black circles) and showed a sustained decrease with dopamine application. I_*K*_ was measured at the end of 40 ms for the voltage step to +20 mV (dark red circles) and remained constant throughout dopamine perfusion. P21 calyx, male. Electrode solution contained K^+^ and the external solution was L-15.

Voltage-dependent Na^+^ channels are a target for neuromodulation in many cell types. In cochlear afferent neurons transient Na^+^ currents are decreased by the application of dopamine (Sun and Salvi, 2001; Valdes-Baizabal et al., 2015). Dopamine may be released from cochlear efferent fibers that terminate on afferent terminals close to inner hair cell synapses (Oestreicher et al., 1997; Ruel et al., 2006). Since dopamine is also a candidate efferent transmitter in the vestibular system (Drescher et al., 2010; Lee and Jones, 2017), we tested its effect on voltage-gated currents in calyces isolated from cristae. Figure 1A shows a series of whole cell currents in response to depolarizing voltage steps before, during and after dopamine application in a P24 calyx. The electrode solution was K^+^-based and therefore both transient Na^+^ currents and outward K^+^ currents were evoked at potentials above ∼ –60 mV. Na^+^ currents were notably reduced during extracellular perfusion of 100 μM dopamine and recovered following return to control extracellular solution. Peak I_*NaT*_ in this cell was decreased during dopamine application at most voltage steps and recovered following washout as shown in the current-voltage plot (Figure 1B).

To test if dopamine affected voltage-dependent outward K^+^ currents in isolated calyces, the potassium current (I_*K*_) was measured during perfusion of 100 μM dopamine in a P21 isolated calyx (Figure 1C). The standard voltage protocol was applied at intervals throughout. Dopamine was applied following stabilization of currents in control conditions, since we previously observed that Na^+^ currents in calyces often increase in size, or run up, during the first few seconds after membrane break through to whole cell recordings (Meredith et al., 2011; Meredith and Rennie, 2018). The peak I_*NaT*_ was measured at steps to –40 mV (black circles) and decreased markedly in the presence of dopamine shortly after the perfusion onset. K^+^ current amplitude was measured at the end of 40 ms voltage steps to +20 mV and remained relatively unchanged throughout the course of dopamine application. In a group of calyces (ages P21-27) mean I_*K*_ was 2,161 ± 1,501 pA in control conditions, which was not significantly different from the mean value of 1,976 ± 1,454 pA in dopamine (mean ± SD, *n* = 6, paired *t*-test, *P* = 0.066). These experiments suggest that the effect of dopamine is specific to Na^+^ currents in calyx terminals.

**FIGURE 2:**
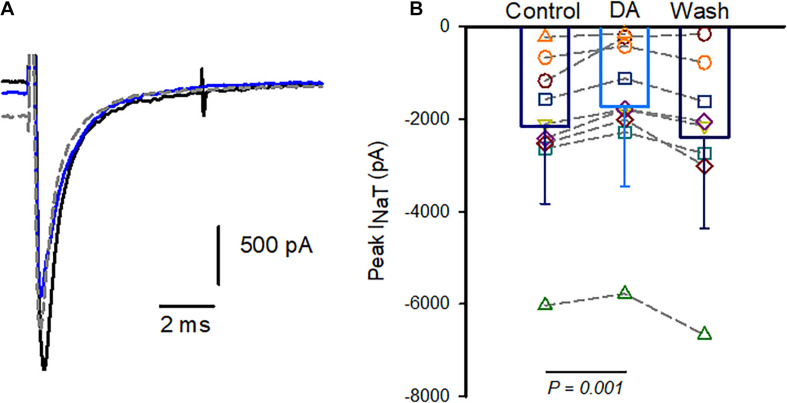
Dopamine reversibly reduces I_*NaT*_ in isolated calyx terminals. **(A)** Whole cell currents in an isolated calyx in response to a voltage step from –90 to –40 mV following a prepulse to –130 mV. Control Na^+^ current (black), current in 100 μM dopamine (blue) and current during washout (gray dashed line) are shown. P22 calyx, female. **(B)** Pooled data show peak currents in individual cells and bars indicate mean I_*NaT*_ in control, in response to 100 μM dopamine (DA) and during washes. Peak currents were measured at voltage steps to –40 mV and averaged –2,151 ± 1,685 pA in control; and –1,732 ± 1,718 pA (mean ± SD, *n* = 9 cells) in dopamine. Recovery during wash was measured in 8 cells since 1 cell was lost prior to wash (P21-27 calyces, 5 K^+^-filled cells and 4 Cs^+^-filled cells).

Having confirmed that K^+^ currents were not significantly affected by dopamine, we performed additional experiments using Cs^+^ in the patch electrode solution to inhibit voltage-dependent K^+^ currents. In the experiments with internal Cs^+^ outward currents were greatly reduced (mean 518.5 ± 241.3 pA at + 20mV, n = 11 cells). The effect of dopamine was tested in four calyces and perfusion with 100 μM dopamine reversibly reduced peak I_*NaT*_ at the test step of –40 mV as shown for one cell in Figure 2A. The effect on Na^+^ current amplitude following dopamine exposure and subsequent washout is shown for cells with either K^+^ or Cs^+^ as the major cation in the patch electrode solution in Figure 2B. Peak Na^+^ currents were measured at –40 mV and averaged –2,151 ± 1,685 pA in control; following dopamine perfusion mean peak current was reduced to –1,732 ± 1,718 pA (*n* = 9 cells). The decrease in amplitude was significant and a partial or complete washout was observed in all but two cells on return to normal extracellular solution (Figure 2B).

### Mechanisms Underlying Na^+^ Current Response to Dopamine

**FIGURE 3:**
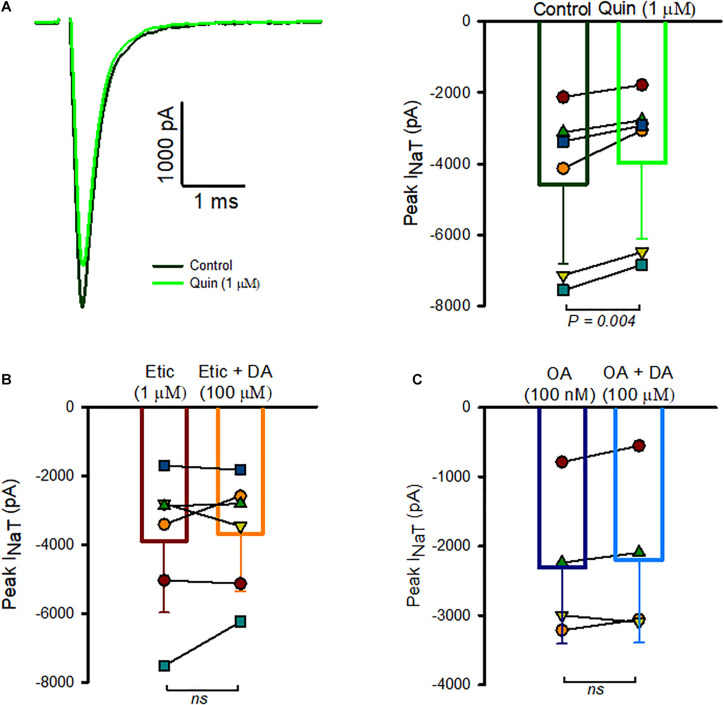
Role of D2-like receptors in I_*NaT*_ inhibition. **(A)** Whole cell currents in an isolated calyx in control (black trace) and following quinpirole perfusion (green trace) are shown in response to a voltage step to –40 mV (left panel). P23 calyx, male. Box plot indicates the response of 6 calyces to quinpirole (right panel). Mean I_*NaT*_ control was –4,573 ± 2,247 pA, mean I_*NaT*_ in quinpirole was –3,978 ± 2,132 pA, *n* = 6, ages P19-28, *P* = 0.004). **(B)** Na^+^ currents were measured in the presence of 1 μM eticlopride, followed by perfusion of 1 μM eticlopride and 100 μM dopamine. Peak Na^+^ currents averaged –3,895 ± 2,083 pA in eticlopride which was not significantly different from peak currents in eticlopride and dopamine (–3,680.2 ± 1,679 pA, *n* = 6, *P* = 0.985). **(C)** Summary of the effect of okadaic acid (OA) in reducing the response of I_*NaT*_ to DA. Mean peak current measured at voltage steps to –40 mV was –2,268 ± 1,170 pA in the presence of internal OA and –2,270 ± 1,061 pA following dopamine perfusion (*n* = 4 cells, all P23). In 1 cell, the initial dopamine perfusion resulted in a reduction of I_*NaT*_, but following a wash and return to control the cell no longer responded to dopamine application. Data from the second perfusion were used in this case.

We next investigated the possible involvement of dopamine receptor subtypes. Dopamine receptors are G-protein-coupled receptors (GPCRs) and five receptor subtypes (D1-D5) have been described (Missale et al., 1998). Based on the effects on adenylyl cyclase activity, receptor subtypes fall into two classes: the D1-like receptor group includes D1 and D5 and D2-like receptors include D2, D3, and D4. Type I spiral ganglion neurons have been shown to express D1, D2, D4, and D5 receptor subtypes (Karadaghy et al., 1997; Inoue et al., 2006; Maison et al., 2012). Immunohistochemical work has provided evidence for D1 and D2 receptors in vestibular epithelia with D2-like immunoreactivity associated with calyx terminals in rat saccule (Drescher et al., 2010). We therefore tested for the presence of dopamine-receptor mediated responses in calyx afferents using quinpirole, an agonist selective for D2 receptors. We found that perfusion with extracellular solution containing 1 μM quinpirole produced a mean reduction of Na^+^ current of 14.2 ± 6.3% (*n* = 6, Figure 3A). Although quinpirole decreased I_*Na*__*T*_ amplitude, we noted that compared to dopamine the effect of quinpirole was smaller and typically required several minutes of perfusion before the reduction in peak Na^+^ current occurred. To further test for D2-like receptor involvement, we used the D2 receptor antagonist eticlopride and assessed its effect on responses to dopamine (Figure 3B). Eticlopride (1 μM) was first applied to cells followed by co-application of 1 μM eticlopride and dopamine (100 μM). There was no significant difference in peak I_*Na*__*T*_ between the two different conditions, suggesting that antagonism of D2 receptors by eticlopride prevented D2 receptor activation by dopamine.

Based on known actions of dopamine on the phosphorylation state of Nav channels in other cell types (Cantrell and Catterall, 2001), we hypothesized that dopamine may modulate Na^+^ current through a change in the phosphorylation state of Na^+^ channels and inhibition of phosphatase might be expected to produce an inhibition of Na^+^ currents analogous to activation of dopamine receptors. We therefore tested the effect of okadaic acid, a known inhibitor of phosphatase 1 and 2A, by perfusing cells with extracellular 100 nM okadaic acid. However, we saw no consistent change in amplitude of I_*Na*_ in 3 cells tested with external perfusion of okadaic acid. Next we studied the effect of including 100 nM okadaic acid in the patch electrode solution followed by extracellular dopamine perfusion (Figure 3C). We measured peak I_*NaT*_ several minutes following membrane breakthrough into the whole cell mode and subsequently began perfusion of dopamine. In these conditions only a small non-significant change in Na^+^ current amplitude occurred following application of 100 μM dopamine (Figure 3C), strongly suggesting that intracellular okadaic acid prevented the action of dopamine on Na^+^ channel activity.

### I_*NaR*_ Is Prevalent in Calyces in Slices, but Is Rarely Observed in Isolated Calyces

**FIGURE 4:**
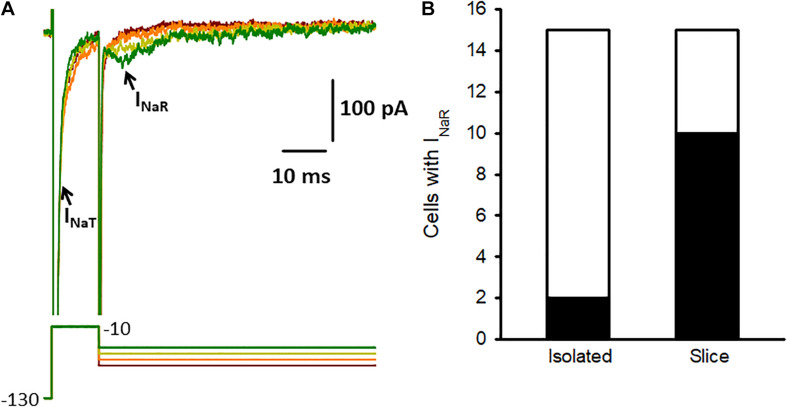
Na^+^ current characteristics in isolated calyces. **(A)** Na^+^ currents (upper) recorded from an isolated P17 calyx using the voltage protocol shown below. From a holding potential of –90 mV the membrane was stepped to –130 mV for 10 ms and then stepped for 10 ms to –10 mV (complete duration of protocol not shown). The protocol resulted in large transient currents (I_*NaT*_, truncated for clarity) at –10 mV, which activated and inactivated rapidly. The membrane was then repolarized to potentials between –75 and –45 mV (10 mV increments shown). Small resurging inward currents (arrow) with characteristics of I_*NaR*_ were seen following repolarizing steps to –35 mV and –45 mV and strongly resembled I_*NaR*_ previously recorded from calyces in slices (Meredith and Rennie, 2020). **(B)** I_*NaR*_ was seen in most calyces in crista slices (10 of 15 cells tested at age P13-15), but was rarely observed in isolated calyces (2 of 15 cells tested, age range P15-33). Electrode solution for these recordings contained Cs^+^ and the HEPES-based external solution contained 120 mM Na^+^.

In previous recordings from mature calyces in crista slices we found that transient sodium currents (I_*NaT*_) were abolished by tetrodotoxin (TTX) (Meredith and Rennie, 2018). We subsequently identified resurgent (I_*NaR*_) and persistent currents (I_*NaP*_), which occurred frequently, but contributed much smaller components of the TTX-sensitive Na^+^ current than I_*NaT*_ (Meredith and Rennie, 2020). Dopamine was reported to modulate only the transient Na^+^ current component in pyramidal neurons (Maurice et al., 2001), but we wondered if dopamine might influence resurgent currents in vestibular calyces. I_*NaR*_ is a distinctive current evoked by transient membrane depolarizations and arises from an open channel block state in certain types of Nav channel (Lewis and Raman, 2014). Calyces dissociated from vestibular epithelia also express TTX-sensitive I_*NaT*_ (Rennie and Streeter, 2006; Meredith et al., 2011), but I_*NaR*_ has not been reported previously in isolated calyces. Given that resurgent current (I_*NaR*_) was observed in greater than half of calyces studied in crista slices at ages P13 and older (Meredith and Rennie, 2020), we tested for the presence of I_*NaR*_ in solitary calyces using voltage protocols designed to maximize I_*NaR*_. The membrane was stepped briefly from –130 to –10 mV to produce activation and inactivation of I_*NaT*_ and then to a series of potentials to repolarize the membrane and evoke I_*NaR*_ (Figure 4A). We discovered that although I_*NaT*_ was present in all isolated calyces tested at ages P15-33 (*n* = 15), I_*NaR*_ was only detected in 2 out of 15 isolated calyces (Figure 4B). Peak I_*NaR*_ in the 2 isolated calyces was small and did not exceed 80 pA in amplitude. This contrasts with our data from calyces in crista slices, where I_*NaR*_ was seen in the majority of calyces and mean peak amplitude exceeded 125 pA at the start of the third postnatal week (Meredith and Rennie, 2020). Nav1.6 channels can underlie transient and resurgent Na^+^ currents and a selective Nav1.6 channel blocker, 4,9 anhydro-TTX, reduced I_*NaT*_ and I_*NaR*_ in both cochlear and vestibular afferents (Browne et al., 2017; Meredith and Rennie, 2020). It remains to be determined whether dopamine can modulate resurgent currents in inner ear primary afferents.

## Discussion

### Dopaminergic Modulation of I_*NaT*_ in Calyx Terminals

In this paper we show for the first time that transient Na^+^ currents in rodent vestibular afferents are modulated by dopamine. Dopamine significantly and reversibly reduced transient Na^+^ currents in acutely isolated calyces. The effect of dopamine was mimicked by quinpirole and inhibited by eticlopride suggesting the presence of D2-like dopamine receptors on calyx-bearing afferents. In contrast, outward K^+^ currents in calyces were not affected by dopamine application.

### Dopaminergic Signaling in the Inner Ear

Dopamine receptors have been classified as either D1-like and D2-like groups based on their biochemical and pharmacological profiles (Missale et al., 1998). Both types are GPCRs and cloning studies have demonstrated five dopamine receptor subtypes (D1–D5). In the cochlea the presence of D1, D2, D4, and D5 receptor subtypes have been reported in type I spiral ganglion neurons (Karadaghy et al., 1997; Inoue et al., 2006; Maison et al., 2012). In cellular studies on isolated and cultured cochlear afferents, dopamine reduced I_*Na*__*T*_ in spiral ganglion neurons of the rat cochlea (Valdes-Baizabal et al., 2015). Na^+^ channels can be modulated through phosphorylation by cAMP-dependent protein kinase A or by protein kinase C (Cantrell and Catterall, 2001). In spiral ganglion neurons both D1- and D2-like receptor subtypes were implicated in dopaminergic modulation of Na^+^ currents. The inhibitory effect of dopamine on I_*NaT*_ was mediated via two separate GPCR pathways, the D1 receptor pathway involved adenylyl cyclase/cAMP/protein kinase A and the D2 receptor pathway was dependent on protein kinase C (Valdes-Baizabal et al., 2015).

In vestibular calyces we found that application of the D2 agonist quinpirole reduced I_*NaT*_ and the D2 antagonist eticlopride blocked the response of calyces to dopamine, strongly suggesting that dopamine modulation occurs via D2 receptors. Phosphatase inhibition by okadaic acid also prevented the effect of dopamine on I_*NaT*_ suggesting phosphorylation involvement. However, a potential role for D1 receptors at the calyx synapse has not yet been ruled out. D1 and D2-like immunoreactivity was reported in vestibular epithelia at the level of vestibular hair cells and afferents in fish and rodent otolith organs and D2 receptors were associated with calyx terminals (Drescher et al., 2010). In multiunit recordings from frog semicircular canal vestibular afferents, dopamine decreased background action potential firing rate and the effect was mimicked by application of both D1 and D2 agonists (Andrianov et al., 2009). More recently dopamine signaling has also been linked to seasonal variations in midshipman fish saccule (Perelmuter et al., 2019). In the cochlea dopamine may have a direct effect on afferent terminals (Sun and Salvi, 2001; Valdes-Baizabal et al., 2015) and also an indirect effect on inner hair cells through inhibition of hair cell glutamate release at the afferent synapse (Wu et al., 2020). Through a decrease in Na^+^ current amplitude and action potential firing rate, the activation of dopamine receptors may reduce excitotoxicity at inner hair cell synapses. Whether dopamine within efferent neurons could undergo dynamic regulation and also serve a neuroprotective role in the vestibular system remains to be determined.

### Dopaminergic Modulation of Other Conductances in Vestibular Afferents

Voltage-dependent K^+^ currents in calyces were not increased or decreased by dopamine application in this study. However, the second messenger cAMP modulates the mixed cation hyperpolarization-activated current (I_*h*_) in auditory and vestibular afferents, where it acts to shift the activation to more positive potentials and increase the degree of current available around the resting potential (Yi et al., 2010; Almanza et al., 2012; Meredith et al., 2012; Horwitz et al., 2014; Ventura and Kalluri, 2019). It is unclear if neurotransmitters act to increase I_*h*_ via cAMP within afferents *in vivo*, but it is plausible that in addition to modulation of the Na^+^ current, dopamine could impact I_*h*_ via a cAMP-dependent signaling pathway. Dopamine has been shown to modulate I_*h*_ in chemosensory neurons of the carotid body, where it acts to decrease firing through a hyperpolarizing shift in I_*h*_ (Zhang et al., 2018). Further examination of the effects of dopamine and associated signaling pathways on other voltage-dependent currents in inner ear afferents could uncover additional modulatory pathways for this candidate efferent transmitter.

### Na^+^ Current Characteristics in Isolated Calyx Terminals

Voltage-gated Na^+^ channels typically produce rapid and transient currents (I_*NaT*_) that mediate the upward phase of the action potential. In addition to I_*NaT*_, resurgent (I_*NaR*_) and persistent Na^+^ currents also manifest in certain cell types (Lewis and Raman, 2014). Dopamine has been shown to differentially modulate Na^+^ current components in pyramidal neurons isolated from the prefrontal cortex, where it inhibited transient but not persistent Na^+^ currents (Maurice et al., 2001). Large and fast activating, rapidly inactivating I_*NaT*_ are present in all mature vestibular afferent calyx terminals in gerbil crista, whereas much smaller resurgent and persistent Na^+^ currents are present in a subset of afferents and all three Na^+^ current components are blocked by tetrodotoxin (Meredith and Rennie, 2018, 2020). Nine distinct Na^+^ channel α subunits have been identified (Nav1.1-1.9) and several of these are expressed in vestibular ganglion neurons (Liu et al., 2016). Nav1.6 channels are present in both cochlear and vestibular afferents where they contribute to transient and resurgent Na^+^ currents (Browne et al., 2017; Meredith and Rennie, 2020). A specific action of dopamine on Nav1.6-mediated currents in inner ear afferents remains to be determined. I_*NaR*_ and I_*NaT*_ were detected together in the majority of mature calyx terminals in crista slices (Meredith and Rennie, 2020). In contrast, I_*NaT*_ was present in all isolated calyces studied here, but I_*NaR*_ was rarely observed. In both vestibular calyces and spiral ganglion neurons I_*NaR*_ expression increased with postnatal development suggesting a role for this current in afferent firing in adult inner ear epithelia (Browne et al., 2017; Meredith and Rennie, 2020). Navβ4 subunits, which may be required for I_*NaR*_ expression (Lewis and Raman, 2014), were detected in vestibular epithelia (Liu et al., 2016) and immunolocalized to the heminode and nodes of Ranvier in cochlear afferent neurons (Browne et al., 2017). Although isolated calyces express several voltage-dependent currents including large I_*NaT*_, I_*h*_ and a variety of K^+^ currents (Hurley et al., 2006; Rennie and Streeter, 2006; Dhawan et al., 2010; Meredith et al., 2011, 2012; Meredith and Rennie, 2015), these solitary calyx terminals for the most part lack axons and could be deficient in endogenous factors needed for I_*NaR*_ generation. Spike generation likely occurs in afferent terminals at the initial unmyelinated segment close to hair cells (Hossain et al., 2005; Lysakowski et al., 2011), but the precise location of Na^+^ channel subtypes remains to be determined.

### Efferent Mechanisms in the Inner Ear

Vestibular hair cells and inner hair cells make synapses with afferent terminals to convey sound and balance information to the brain as action potentials, but the auditory and vestibular systems also receive extensive efferent input from the brain. Outer hair cells and type II hair cells are contacted directly by efferent terminals, but efferent fibers also terminate on unmyelinated vestibular calyx terminals and auditory afferents postsynaptic to inner hair cells. The strategic placement of efferent synapses in the initial segment zone of the axon and calyx outer face could allow modulation of afferent firing close to the site of action potential initiation through receptor-mediated influence on current components mediated through voltage-gated ion Na^+^ channels.

Gerbil cristae receive substantial efferent innervation with distinct efferent groups projecting to ipsilateral and contralateral cristae (Purcell and Perachio, 1997). Although acetylcholine is considered a major efferent neurotransmitter, as shown in gerbil not all vestibular efferent neurons are cholinergic (Perachio and Kevetter, 1989). The activation of efferent fibers has been shown to have complex effects (both excitatory and inhibitory) on action potential firing rate in vestibular afferents. Acetylcholine released from efferent terminals can act via both nicotinic and muscarinic receptor signaling pathways (Holt et al., 2015, 2017; Lee et al., 2017; Poppi et al., 2020). Ca^2+^ influx through α9-containing nicotinic receptors activates SK channels producing a hyperpolarization of type II vestibular hair cells and a decrease in membrane resistance and hair cell sensitivity (Poppi et al., 2018, 2020). A similar mechanism exists in the cochlea where the efferent-mediated release of acetylcholine inhibits outer hair cell function through α9 receptors (Fuchs and Lauer, 2019). Conversely, acetylcholine can result in excitation through muscarinic receptor activation which inhibits M-like K^+^ currents in calyx afferents of turtle and rat crista (Holt et al., 2017; Ramakrishna et al., 2021). Other transmitters may also contribute to efferent transmission (Lee and Jones, 2017). In rat crista, GABA was recently shown to have an excitatory effect on calyx firing through GABA_*B*_ receptor-mediated inhibition of K^+^ channels (Ramakrishna and Sadeghi, 2020). Therefore several studies have suggested efferent-mediated modulation of K^+^ currents in vestibular hair cells and afferents, but direct effects of potential neuromodulators on Na^+^ channels linked to firing have not been reported until now. Our data suggest dopamine release could impact firing in vestibular afferents through activation of D2 receptors and associated intracellular signaling mechanisms resulting in Na^+^ channel inhibition within calyx terminals. As described in cochlear afferents, dopamine-mediated reduction of I_*NaT*_ would result in a decrease in firing in vestibular calyx-bearing neurons.

Dopaminergic efferents are present in the cochlea and may also be present in vestibular epithelia. Tyrosine hydroxylase is required for the synthesis of dopamine and Drescher et al. (2010) identified thin tyrosine hydroxylase-containing nerve fibers in otolith epithelia including the rat saccule and utricle and mouse utricle. These presumed efferent fibers terminated on vestibular hair cells and also on calyces. In addition, D1 (D1A) and D2 (D2L) receptor immunoreactivity was seen within hair cells and neurons supporting a role for dopamine as an efferent neurotransmitter in the vestibular system (Drescher et al., 2010).

## Concluding Remarks

Dopamine may operate as a neuroactive substance at the hair cell/afferent synapse in mammalian auditory and vestibular systems. Through a reduction in sodium channel activity via changes in phosphorylation, dopamine released from efferents may reduce action potential firing in both auditory and vestibular afferents. In the cochlea, dopamine appears to exert its inhibitory effect on afferent firing via two distinct dopaminergic signaling pathways. Our data support a role for D2 receptors in Na^+^ current inhibition and the involvement of other dopamine receptor subtypes remains to be elucidated. Disruption to dopaminergic efferent pathways could be associated with auditory and vestibular dysfunction.

## Data Availability Statement

The raw data supporting the conclusions of this article will be made available by the authors, without undue reservation.

## Ethics Statement

The animal study was reviewed and approved by University of Colorado Denver Institutional Animal Care and Use Committee.

## Author Contributions

FM and KR designed the experiments, performed the research experiments, analyzed the data, and prepared the figures. KR wrote the first draft of the manuscript. Both authors contributed to the article and approved the submitted version.

## Conflict of Interest

The authors declare that the research was conducted in the absence of any commercial or financial relationships that could be construed as a potential conflict of interest.

## Publisher’s Note

All claims expressed in this article are solely those of the authors and do not necessarily represent those of their affiliated organizations, or those of the publisher, the editors and the reviewers. Any product that may be evaluated in this article, or claim that may be made by its manufacturer, is not guaranteed or endorsed by the publisher.
