# Erratum: Dopaminergic Inhibition of Na^+^ Currents in Vestibular Inner Ear Afferents

**DOI:** 10.3389/fnins.2022.866989

**Published:** 2022-03-15

**Authors:** Frontiers Production Office

**Affiliations:** Frontiers Media SA, Lausanne, Switzerland

**Keywords:** calyx, semicircular canal, crista, hair cell, sodium channel

Due to an editorial mistake, the acknowledgment for the participation of a third reviewer is missing. The acknowledgment is the following: ‘Selina Baeza-Loya, University of Chicago, United States, in collaboration with reviewer RE'. The publisher apologizes for this mistake.

The original article has been updated.

## Publisher's Note

All claims expressed in this article are solely those of the authors and do not necessarily represent those of their affiliated organizations, or those of the publisher, the editors and the reviewers. Any product that may be evaluated in this article, or claim that may be made by its manufacturer, is not guaranteed or endorsed by the publisher.

